# Maternal mental health is associated with children's frequency of family meals at 12 and 24 months of age

**DOI:** 10.1111/mcn.13552

**Published:** 2023-08-18

**Authors:** Christine Helle, Elisabet R. Hillesund, Nina Cecilie Øverby

**Affiliations:** ^1^ Department of Nutrition and Public Health, Faculty of Health and Sport Sciences University of Agder Kristiansand Norway

**Keywords:** family meals, infant and child nutrition, maternal mental health, public health

## Abstract

Diet during the child's first years is important for growth and development. In toddlerhood, higher diet quality is reported among children eating meals together with family. Although previous literature has documented several associations between maternal mental health and early child feeding practices, less is known about the relationship between maternal mental health and child frequency of shared family meals. This study explores associations between maternal symptoms of anxiety and depression, measured by The Hopkins Symptoms Checklist (SCL‐8), and toddler participation in family meals. We used cross‐sectional data from the Norwegian study *Early Food for Future Health*, in which participants responded to questionnaires at child age 12 (*n* = 455) and 24 months (*n* = 295). Logistic regression was used to explore associations between maternal mental health and child having *regular* (≥5 per week) or *irregular* (<5 per week) family meals (breakfast and dinner), adjusting for relevant child and maternal confounding variables. Children of mothers with higher scores of anxiety and depression had higher odds of *Irregular family meals* at both timepoints; (OR: 2.067, *p* = 0.015) and (OR: 2.444, *p* = 0.023). This is one of few studies exploring associations between maternal mental health and child frequency of shared family meals in early childhood, a period where the foundation for life‐long health is shaped. Given the high prevalence of mental ailments and disorders, these findings are important and may inform future public health interventions. Further exploration of this relation is needed, including longitudinal research to test predictive associations and qualitative studies to increase insight and understanding.

## Key messages

- Previous literature has documented several associations between maternal mental health and early child feeding practices, but less is known regarding the relationship between maternal mental health and toddlers' frequency of shared family meals.
- We found that maternal symptoms of anxiety and depression was positively associated with higher odds of child having irregular family meals in the two first years.
- Our findings support the importance of early identification of maternal symptoms of anxiety and depression to help mothers having positive interactions around food and eating with their toddlers.
- Given the health benefits of the family meal as a setting for child diet and dietary behaviours, this study's results may have major implications by informing design of future public health interventions.

## Graphical abstract

This study is among the first exploring associations between maternal mental health and toddler participation in family meals. Children of mothers with more symptoms of anxiety and depression had higher odds of irregular family meals at 12 and 24 months. Our findings have importance by informing future public health interventions.

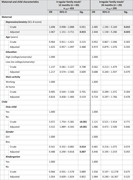

## 1 INTRODUCTION

The child's nutritional environment during the first 1000 days of life is highly important for later health outcomes (Hanson et al., 2015; Schwarzenberg et al., 2018). During this period, children are totally reliant on their parents concerning *what* and *how* they are served. The ways parents interact with children around food and meals affect children's diet and socialization as well as the development of eating habits (Litterbach et al., 2017; Pearson et al., 2009). As health behaviours established in early childhood tend to track over time (Mikkilä et al., 2005; Montaño et al., 2015; Savage et al., 2007), these two first years represent a unique window of opportunity to promote beneficial, and prevent the formation of detrimental, eating habits.

During the last few decades, the family meal has received attention in research and is now recognized as an important component of nutritional health promotion for children (Martin‐Biggers et al., 2014). A meta‐analysis that included more than 180,000 children and adolescents (3–17 years), showed that children who participated in family dinners at least three times a week had better diet quality and a lower likelihood of obesity than those who rarely participated in shared dinners (Hammons & Fiese, 2011). Another recent meta‐analysis including 57 studies and more than 200,000 participants, reported a significant relationship between frequent family meals and better nutritional health in younger and older children across countries and socioeconomic groups (Dallacker et al., 2018). A systematic review of 81 studies including children and adolescents (2–18 years), showed that an increasing number of family meals was associated with a healthier diet and nutrient intake and less overweight and obesity (Martin‐Biggers et al., 2014). Some of the included studies also indicated positive relationships with outcome measures such as belonging, self‐esteem, language development and academic performance. Similar findings have also been reported for the youngest age groups. A recent review including infants and toddlers (0–3 years) found that sharing meals as a family was associated with more nutrient‐dense food intake, less fussy eating, and higher levels of food enjoyment as well as health benefits (Verhage et al., 2018). Results from a previous study by our research group showed that being fed in the context of family meals was associated with a more favourable diet in infants as young as 12 months of age (Hillesund et al., 2021).

The family meal constitutes a social setting with the potential to shape children's eating routines and behaviours as well as taste preferences from an early stage. When eating together, parents can model healthy eating and expose children to varied and healthy foods (Yee et al., 2017). However, family meals also provide opportunities for communication, sharing of values and family bonding. Meals have a social function when eating in the company of other people. It is well documented that social contact is of great importance for mental health (Fasihi Harandi et al., 2017), which may explain the existing evidence suggesting that family meals promote emotional well‐being and protect against later health risks (Utter et al., 2018).

Previous research has documented that social disparities exist in children's dietary intake and diet quality, with low maternal education conferring the highest risk (Rashid et al., 2020). This also applies to children's participation in family meals, which are more frequent in families with higher education and socioeconomic status (Neumark‐Sztainer et al., 2013; Vik et al., 2016). Other sociodemographic characteristics that have been associated with more frequent or positive aspects of family meals include gender (boys) (Hillesund et al., 2021; Neumark‐Sztainer et al., 2003, 2013) and living in a two‐parent household (Larson et al., 2013; Pearson et al., 2009).

With family meals being an influential and important arena for children's development of a healthy diet as well as emotional well‐being, identifying potential barriers that may prevent the child from participating in regular family meals is important. From previous research we know that maternal mental health may impact different aspects of the child's diet and eating behaviors. Maternal symptoms of anxiety and depression has been associated with a lower extent of breastfeeding (Ystrom et al., 2008) and a less wholesome and a more unhealthy child diet (Ashaba et al., 2015; Ystrom et al., 2012). Maternal symptoms of anxiety and depression has further been associated with increased use of less responsive and more intrusive feeding practices, for example, use of control or pressure to make the child eat (Haycraft et al., 2013; Hurley et al., 2008; Ystrom et al., 2008, 2009). Despite these correlations between maternal mental health and child diet and feeding outcomes, less research has explored associations between maternal mental health and child frequency of family meal participation during early childhood. A study including mothers of school‐aged children in the United States, found that when maternal mental health declined, so did the frequency of family meals (Keresztes et al., 2022). Another study among parents of adolescents in the United States, reported that frequent parent‐reported family meals was associated with lower levels of parental depressive symptoms and stress (Utter et al., 2018). In a third study among 164 mothers of 2 to 5‐year‐old low‐income children in the United States, maternal depression was associated with lower maternal control over child eating routines, lower maternal presence when child ate and more negative mealtime practices (McCurdy et al., 2014). To our knowledge, no previous research has examined the relationship between maternal mental health and child's participation in shared family meals during the formative phase that constitutes the first 2 years. In this paper we aim to address this knowledge‐gap by exploring potential associations between maternal symptoms of anxiety and depression and child frequency of family meals at 12 and 24 months of age, adjusting for relevant co‐variates highlighted by previous research.

## 2 METHODS

### 2.1 Study design and participants

This paper presents a secondary analysis of data from the Norwegian randomized controlled trial (RCT) *Early food for future health*. The original RCT was a primary prevention intervention, evaluating a digital dietary intervention targeting parents of 6 to 12‐month‐old children aiming to promote healthy food habits from infancy. In short, the intervention was a website from which parents in the intervention group had access to age‐specific information regarding child diet and food‐related child‐parent interactions. Parents were eligible to participate in the study if they had a 3 to 5‐month‐old infant born after gestational week 38, were literate in Norwegian and responsible for providing food to their infant.

In 2016, a total of 715 mothers completed the baseline questionnaire at child age 5 months (T1). Post‐intervention data were collected at child age 12 months (T2), and follow‐up data at child age 24 months (T3). The results from the original RCT are previously reported elsewhere (Helle et al., 2019a, 2019b). For the current paper we included the data collected at T2 (*n* = 455) and T3 (*n* = 295). All data were collected from web‐based, self‐administered questionnaires.

Informed consent was obtained from all participants on the study's homepage when signing up for study participation. This study was notified to the Norwegian Social Data Services, Data protection Official for Research, 17/08/2015 (http://pvo.nsd.no/prosjekt/4397).

### 2.2 Outcome variables

#### 2.2.1 Family meal frequency

For this study, we included both breakfast and dinner as family meals. In Norway, breakfast and dinner are the most important meals in families with preschool‐age children. The vast majority of Norwegian children attend kindergarten and eat their lunch there, and supper is usually a separate meal for children before bedtime. Eating breakfast and dinner is further associated with more healthy dietary patterns (Ardeshirlarijani et al., 2019; Mahmood et al., 2023; Vik et al., 2016) and more dietary benefits (Hillesund et al., 2021) than the other meal categories.

The frequency of family meals was assessed by parents responding to following two questions: *How often does your child eat breakfast together with the family now?* and *How often does your child eat dinner together with the family now?* Family was defined as at least one adult eating the same meal. Response alternatives were *never/seldom, 1–3 times per week, 4–6 times per week and every day*, with possible scores ranging from 1 *(never/seldom)* to 4 *(always)*. These two variables were combined into a *Family*‐*meal frequency score* with possible scores ranging from 2 *(never/seldom breakfast and dinner)* to 8 *(always breakfast and dinner)*.

A previous systematic review of the effects of family meal frequency on psychosocial outcomes in youth, identified that a frequency of at least five times a week widely denotes a regular practice of family meals (Harrison et al., 2015), and several previous studies have used the same categorization (Agathão et al., 2021; Burgess‐Champoux et al., 2009; Vik et al., 2016). We restricted having a regular family meal practice to eating breakfast and dinner with family five times per week or more to encompass a predictable mealtime‐structure that may guide health‐promoting behaviors and development. As the current study's response‐options did not fully correspond to this suggested cut‐off, we categorized the outcome variable *Regular family meals* as having a score above or equal to seven to obtain the best resemblance. Similarly, we defined a score below or equal to five as the second outcome variable *Irregular family meals*, corresponding to having family meals less than five times a week.

### 2.3 Predictor variables

#### 2.3.1 Maternal mental health

We assessed maternal self‐reported symptoms of depression and anxiety with the short version (SCL 8) of the Hopkins Symptoms Checklist (SCL 90). SCL 8 consists of eight items: four items measuring depression and four items measuring anxiety. SCL 90 is a well‐established psychometric instrument (Derogatis et al., 1973), and the short version used in this study has previously been used in the Norwegian population‐based MoBa study (Tambs & Røysamb, 2014). Questions posed were: *Have you during the last two weeks been bothered by the following?* with four response‐categories ranging from not bothered to very bothered. These response‐categories are rated from 1 to 4, with higher scores reflecting more severe symptoms. The SCL 8 total score was computed by adding the eight item scores and dividing the sum‐score on the number of items. Cronbach's alpha for this scale at child age 12 and 24 months was 0.83, indicating a good internal consistency.

#### 2.3.2 Potential mother and child confounding variables

We included mother and child variables that provided relevant background information or based on previous literature, could have a potential influence on the outcome. Maternal age was computed based on self‐reported birth date. Maternal education was originally mapped by asking *What is your highest completed education*, with response‐options on a 7‐category scale. These were subsequently recoded into *high* versus *low* education *(college/university vs. no college/university*). Civil status was assessed by *What is your civil status now?* Response alternatives were dichotomized into *Married/cohabitant* and *Living alone*. Main daily activity was assessed by: *What is your main activity now?* The 10 response alternatives were dichotomized into *Working* (full time or part time) and *Not working* (all other response alternatives).

Child gender and number of children were self‐reported by the mothers. Attendance in kindergarten was mapped by asking *Where is the child taken care of during the day now?* The answer‐options were dichotomized into *Kindergarten* or *Not kindergarten*.

### 2.4 Statistical analysis

Descriptive statistics was used to present background data of mothers and children with means and standard deviations (SDs) for continuous data and numbers and percentages for categorical data. To assess the associations between maternal mental health and child frequency of family meals at child age 12 and 24 months, we created two multivariable logistic regression models with *Regular family meals* and *Irregular family meals* as the two dependent variables and symptoms of maternal anxiety or depression measured with the SCL‐8 total score as the independent variable. We adjusted for maternal age, education, and main daily activity, as well as for child gender, if siblings, and for kindergarten attendance. All models were adjusted for group allocation (control or intervention) in the original RCT. Preliminary analyses were performed to test for potential violations of multicollinearity between the independent variables in the regression models. The categorical independent variables were treated as continuous in linear regression, and collinearity diagnostics were performed. All variance inflation factors (VIF‐values) were below 10, indicating absence of highly collinear relationships between the predictor variables. Maternal civil status was not included as a co‐variate due to the very low percentage of single mothers in the current study‐sample. Instead, Fisher's exact test was used to explore potential significant associations between maternal civil status and child having *Regular* or *Irregular family meals*. We used a complete case approach in the analyses. All maternal and child predictor variables were included simultaneously and mutually adjusted in the regression models. Lastly, as supplementary analyses we explored the constructs separately for anxiety and depression.

The analyses were done using SPSS 25.0 (IBM Corp). The study size was calculated according to the original randomized controlled trial (Helle et al., 2017). Sample size estimates for the original trial's primary outcomes are previously published (Helle et al., 2019a). Statistical significance was defined as a *p* < 0.05.

## 3 RESULTS

Maternal and child characteristics at child age 12 and 24 months are given in Table 1. The mean SCL 8 scores at child age 12 and 24 months in this sample were 1.4 and 1.3 respectively (possible range 1.00–4.00). This is similar to mean scores reported in previous Norwegian population‐based studies (Hampson et al., 2010; Ystrom et al., 2008). At child age 12 months, maternal age ranged from 20 to 44 years (mean 31.3). Approximately 85% of the participating mothers were highly educated, and the vast majority of mothers were married or cohabitant (97%–98%). The proportion of mothers who were working outside home increased from child age 12 months (69%) to 24 months (74%).

**Table 1:** Maternal and child characteristics at child age 12 and 24 months.

	Child age 12 months (*n* = 455)	Child age 24 months (*n* = 295)
Maternal and child characteristics	Mean (SD) or *n* (%)^a^	Mean (SD) or *n* (%)^a^
Maternal		
Depression/anxiety (SCL‐8‐score)	1.4 (0.4)	1.3 (0.4)
Age (years)	31.3 (4.3)	32.4 (4.2)
Education		
High *(college/university)*	378 (84)	255 (86)
Low *(no college/university)*	71 (16)	40 (14)
Civil status		
Married/cohabitant	443 (97)	288 (98)
Living alone	12 (3)	7 (2)
Main activity		
Working	315 (69)	217 (74)
At home	140 (31)	78 (26)
Child		
Gender		
Girls	226 (50)	149 (50)
Boys	229 (50)	146 (50)
Only child		
Yes	266 (59)	145 (49)
No	189 (41)	150 (51)
Kindergarden		
Yes	173 (38)	280 (95)
No	282 (62)	15 (5)
Family meals		
Regular family meals^b^	270 (60)	171 (58)
Irregular family meals^c^	83 (18)	29 (10)
Not classified as regular or irregular	102 (22)	95 (32)

a Valid percentages for categorical variables and means with SD for continuous variables.

^b^ Having breakfast and dinner with at least one adult eating the same meal five or more times per week.

^c^ Having breakfast and dinner with at least one adult eating the same meal less than five times per week.

The proportion of boys was 50% at both timepoints, which is close to the national average (Statistics Norway, 2016). The proportion of children who were only child decreased from 59% to 49% from child age 12 to 24 months, while the proportion attending kindergarten increased from 38% to 95% during the same time span. Approximately 60% of the mothers reported that their child had regular family meals at 12 and 24 months of age, while 18% and 10%, respectively, reported irregular family meals at the same timepoints. As the current study's response‐options did not fully correspond to the suggested cut‐off for regular family meals as five times or more per week, 22% and 32% of the children were neither classified as having regular nor irregular family meals at 12 and 24 months of age, respectively.

Table 2 presents associations between maternal mental health and child having regular family meals at 12 and 24 months of age. We found no significant associations between maternal mental health and child having regular family meals at either 12 or 24 months of age. However, we found that children having siblings were less likely to have regular family meals compared to children being only child at child age 12 months (OR = 0.584; *p* = 0.013). We further found that children not attending kindergarten were less likely to have regular family meals at both 12 months (OR = 0.617; *p* = 0.024) and 24 months of age (OR = 0.170; *p* = 0.023), respectively.

**Table 2:** Associations between maternal and child characteristics and child having regular family meals at 12 and 24 months of age, OR (95% CI).

Maternal and child characteristics	Regular family meals^a^			Regular family meals^a^		
	12 months (*n* = 270)			24 months (*n* = 171)		
	*n*_tot_ = 455			*n*_tot_ = 295		
	OR	95% CI	Significance	OR	95% CI	Significance
Maternal						
Depression/anxiety (SCL‐8 score)						
Crude	0.921	0.583–1.457	0.726	0.765	0.435–1.347	0.354
Adjusted	0.939	0.579–1.523	0.799	0.721	0.396–1.313	0.285
Age (years)						
Crude	1.029	0.984–1.076	0.212	1.039	0.982–1.098	0.183
Adjusted	1.009	0.959–1.062	0.722	1.042	0.980–1.108	0.193
Education						
High *(college/university)*	1.000			1.000		
Low (no college/university)						
Crude	0.993	0.593–1.664	0.980	1.150	0.588–2.249	0.683
Adjusted	0.944	0.557–1.724	0.944	0.990	0.483–2.027	0.977
Main activity						
Working	1.000			1.000		
At home						
Crude	0.845	0.561–1.270	0.417	1.015	0.601–1.715	0.954
Adjusted	0.945	0.596–1.497	0.809	1.000	0.563–1.779	0.999
Child						
Only child						
Yes	1.000			1.000		
No						
Crude	0.546	0.370–0.805	**0.002**	0.997	0.628–1.583	0.990
Adjusted	0.584	0.382–0.894	**0.013**	1.111	0.674–1.832	0.680
Gender						
Girl	1.000			1.000		
Boy						
Crude	1.334	0.917–1.941	0.132	1.448	0.910–2.303	0.118
Adjusted	1.288	0.872–1.901	0.203	1.535	0.953–2.474	0.078
Kindergarten						
Yes	1.000			1.000		
No						
Crude	0.639	0.435–0.938	**0.022**	0.199	0.044–0.900	**0.036**
Adjusted	0.617	0.405–0.938	**0.024**	0.170	0.037–0.786	**0.023**

*Note*: Bold values indicate statistical significance.

^a^ Having breakfast and dinner with at least one adult eating the same meal five or more times per week.

In Table 3, associations between maternal mental health and child having irregular family meals at 12 and 24 months of age are displayed. We found that children of mothers reporting more symptoms of anxiety and depression were more likely to have irregular family meals at both 12 months of age (OR = 2.067; *p* = 0.015) and 24 months of age (OR = 2.444; *p* = 0.023). When exploring these constructs separately for anxiety and depression in supplementary analyses, we found that the mean scores were slightly higher for depression at both 12 and 24 months of age (not shown), while the associations with irregular family meals were stronger for anxiety at the same two timepoints (Supporting Information: Table 4). At child age 12 months, we found that children with siblings were more likely to have irregular family meals compared to being only child (OR = 3.522; *p* ≤ 0.001), and that boys were less likely to have irregular family meals compared to girls (OR = 0.488; *p* = 0.007).

**Table 3:** Associations between maternal and child characteristics and child having irregular family meals at 12 and 24 months of age, OR (95% CI).

	Irregular family meals^a^			Irregular family meals^a^		
	12 months (*n* = 83)			24 months (*n* = 29)		
	*n*_tot_ = 455			*n*_tot_ = 295		
Maternal and child characteristics	OR	95% CI	Significance	OR	95% CI	Significance
Maternal						
Depression/anxiety (SCL‐8 score)						
Crude	1.698	0.998–2.888	0.051	2.485	1.195–5.169	**0.015**
Adjusted	2.067	1.151–3.711	**0.015**	2.444	1.130–5.286	**0.023**
Age (years)						
Crude	0.964	0.911–1.021	0.215	0.952	0.867–1.046	0.303
Adjusted	1.025	0.957–1.097	0.488	0.973	0.879–1.076	0.593
Education						
High *(college/university)*	1.000			1.000		
Low *(no college/university)*						
Crude	1.137	0.581–2.227	0.708	0.562	0.213–1.479	0.243
Adjusted	1.217	0.574–2.582	0.609	0.688	0.244–1.937	0.479
Main activity						
Working	1.000			1.000		
At home						
Crude	0.905	0.544–1.506	0.701	0.653	0.289–1.472	0.304
Adjusted	0.824	0.458–1.483	0.519	0.724	0.297–1.766	0.478
Child						
Only child						
Yes	1.000			1.000		
No						
Crude	3.072	1.754–5.381	**≤0.001**	1.121	0.521–2.414	0.771
Adjusted	3.522	1.889–6.565	**≤0.001**	1.086	0.472–2.500	0.846
Gender						
Girl	1.000			1.000		
Boy						
Crude	0.541	0.332–0.882	**0.014**	0.481	0.216–1.073	0.074
Adjusted	0.488	0.290–0.818	**0.007**	0.446	0.195–1.019	0.055
Kindergarten						
Yes	1.000			1.000		
No						
Crude	0.966	0.591–1.578	0.889	1.556	0.197–12.278	0.675
Adjusted	1.054	0.609–1.824	0.852	1.984	0.240–16.367	0.525

*Note*: Bold values indicate statistical significance.

^a^ Having breakfast and dinner with at least one adult eating the same meal five or more times per week.

The very low proportion of single mothers made it difficult to explore potential associations between maternal civil status and the dependent variables *Regular* and *Irregular family meals* using logistic regression. Instead, Fisher's exact test was used to explore these associations at child age 12 and 24 months. We found no significant associations between maternal civil status and child having regular or irregular family meals, neither at child age 12 months (*p* = 0.061 and *p* = 0.621, respectively) nor child age 24 months (*p* = 0.328 and *p* = 0.481, respectively).

## 4 DISCUSSION

The present study explores potential associations between maternal symptoms of anxiety and depression and the child's participation in family meals at 12 and 24 months of age. Our results indicate that maternal symptoms of anxiety and depression are associated with a higher risk for child having irregular family meals. We further found that having siblings and being a girl was associated with having irregular family meals at 12 months of age, while being only child was associated with having regular family meals at 12 months of age. Lastly, we found that children attending kindergarten were more likely to have regular family meals at both 12 and 24 months of age compared to children not attending kindergarten.

### 4.1 Frequency of family meals

We found that about 60% of the children were having regular breakfast and dinner with their families at 12 and 24 months of age. Our findings are in line with previous results, however direct comparisons between studies are difficult as most previous studies explore frequency of family meals in older children and adolescents, and children's participation in family meals decreases with increasing age beyond the preschool age (McCullough et al., 2016). A European study of 7716 children aged 10–12 years across eight countries including Norway, found that 35% of the children had regular family breakfast and 76% had regular family dinner (≥5/week) (Vik et al., 2016). In a recent Norwegian report on Health and well‐being of children and young people, about half of the children stated that they eat at least one meal with their family every day, with the highest prevalence among the youngest age groups (University of Bergen, 2020). Our study contributes to the research field with new and important knowledge regarding mealtime habits in the first years of life.

Despite the numerous positive outcomes that have been associated with having frequent family dinners, there is a growing concern that family meals decrease in frequency. As a general trend, family dinner frequency has gone down over the past decades (Neumark‐Sztainer et al., 2013; Walton et al., 2016). Our estimates are probably higher than in the general population as a whole due to the study sample's high proportion of well‐educated mothers. The diet of Norwegian infants and toddlers is regularly followed through large, nationwide surveys (The Norwegian Directorate of Health, 2023). However, these surveys do not include items mapping the child's participation in family meals. In light of the well documented pattern of positive associations between family meals and health benefits in children, questions addressing frequency of family meals among young children should be incorporated into future nationwide surveys to follow trends in prevalence and development.

### 4.2 Maternal mental health and child frequency of family meals

To our best knowledge, this is the first study to report associations between maternal symptoms of anxiety and depression and child participation in family meals during the first 2 years. Our results suggest that maternal symptoms of anxiety and depression could be an obstacle for children having regular family meals in this period. Supplementary analyses revealed that these associations were stronger for anxiety. As mental ailments and disorders are very commonly occurring, our findings may have major public health implications. In Norway, 22% of all women were in contact with the primary health care services in 2020 due to mental health problems (Norwegian Institute of Public Health, 2023). This is in line with figures from other countries. A systematic review and meta‐analysis on the global prevalence of common mental disorders across both high, low and middle income countries, reported that on average one in five adults (17.6%) experienced a common mental disorder within the past 12 months and 3 in 10 (29.2%) across their lifetime (Steel et al., 2014). Mothers of infants may be particularly vulnerable as postpartum depression (PPD) affects between 7% and 13% of women who give birth (Stewart & Vigod, 2016). Although most cases resolve within a few months with treatment, 24% of women diagnosed with PPD are still depressed 1 year after giving birth and 13% after 2 years (Stewart & Vigod, 2019).

Having symptoms of anxiety and depression may affect the interplay between mother and child, and influence mothers' ability to promote healthy environment and behaviors for their children. Common maternal mental disorders such as depression and anxiety have been identified as risk factors for impaired child development and poor infant growth and cognitive development (Atif et al., 2015; Liu et al., 2017; Tuovinen et al., 2018). Potential mechanisms may be related to less maternal sensitivity or responsiveness, which is associated with higher rates of negative emotional expressions and less predictable and consistent parenting (Lovejoy et al., 2000). Infants and toddlers are more susceptible to the effects of maternal symptoms of depression and anxiety, which may have long‐term consequences and persist into adolescence (Sanger et al., 2015).

Lack of initiative and feeling tired are common symptoms of depression, in addition to reduced self‐esteem and feelings of guilt or inferiority (NHS, 2019, December 10). Having these difficulties may be a hindrance to orchestrating family meals, which require both time and effort for planning and preparation of foods. The review by Verhage et. al. reported that mothers perceived the family meal as a valuable moment, however different mealtime stressors like the child's behaviour (e.g., picky or stubborn) and possible mess and difficulties in planning the family meal, were reported as obstacles leading to fewer family meals (Verhage et al., 2018). Mental health problems may heighten the threshold for coping with the child's negative emotions and activate feelings of stress and worry. It is therefore likely that meals and meal preparation can be perceived as less manageable by mothers with symptoms of anxiety or depression. A previous study reported that when maternal mental health declined, there were fewer family meals as well as greater use of nonrecommended child feeding practices and greater sugar‐sweetened beverage supplies (Keresztes et al., 2022). For frequency of family meals and use of nonrecommended child feeding practices, the associations were moderate to strong. As an extension of these findings, a previous paper from our research group reported that having family meals every day was associated with positive parental feeding practices, while having family meals less often was associated with negative feeding practises (Øverby et al., 2020). Taken together, these findings indicate that maternal symptoms of anxiety and depression may have implications for both the quantity and the relational feeding quality of the family meal.

### 4.3 Other findings

There is limited existing knowledge regarding how parental food choices differ between boys and girls during early childhood. In our study, we found that girls were more likely to have irregular family meals compared to boys at 12 months of age. To our knowledge, this has not been previously reported. A previous study exploring the cross‐sectional relationship between frequency of family meals and child diet at 12 months of age, found that the positive association between frequency of family meals and vegetable intake, home‐made dinners and drinking water only was confined to boys (Hillesund et al., 2021). Also, studies on infants have found that boys tend to be introduced to solid food earlier than girls (Grote et al., 2011; Helle et al., 2018), and that male infants were more likely to be weaned because of hunger and needing food while female infants were more likely to be introduced to solid foods in an attempt to settle behaviour (Brown & Rowan, 2016). Taken together, one may speculate whether mothers, consciously or unconsciously, have different perceptions about their child's nutritional needs linked to gender and a greater focus on boys' nutrition and eating compared to girls'. These early childhood gender‐differences in shared family meals and parental feeding practices should be further explored in future research.

Well‐known barriers to family meals include lack of time and conflicting schedules (Jones, 2018; Martin‐Biggers et al., 2014; Quick et al., 2011). Family meal frequency can easily be impacted by time issues associated with younger children and childcare routines. Parents who are rushing home from work and picking up children from daycare, may find it hard to plan, prepare and serve a family dinner for their families. However, we found that attending kindergarten was positively associated with having regular family meals at both 12 and 24 months of age. One could speculate whether these fixed every day routines facilitate an every day structure that incorporates regular family meals to a greater extent. In Norway, dual‐earner families are common and the proportion of mothers working full or part time outside the home is almost 80% (Egeland et al., 2021). The proportion of children between 1 and 2 years attending kindergarten is 88% (Statistics Norway, 2023), and knowledge of how this may affect the family's every day life is important. Our findings do not suggest that attending kindergarten negatively affects the regularity of shared family meals among the youngest children.

Why having siblings was associated with less frequent family meals at 12 months of age but not at 24 months of age, was also an unexpected result which may be difficult to interpret. However, 1‐year old children require more hands‐on feeding strategies, and parents may perceive shared family meals with children of this age as extra stressful or inconvenient when they also have older siblings to look after. It may be that parents in this situation make different meals or separate feeding schedules for the youngest children from themselves and the rest of the family. A qualitative study exploring parent's perceptions about the challenges of preparing and executing family meals for young children found that developmental challenges, like the child being messy at the dinner‐table or that children at dissimilar stages were eating different foods, was a barrier to shared family meals (Quick et al., 2011).

### 4.4 Strengths and limitations

Our study has some important strengths. To our best knowledge, this is among the first studies to explore associations between maternal mental health and child frequency of shared family meals in the child's two first years. Although mental health difficulties and disorders are common in the population, less is known regarding whether this may impact mother's ability to create health‐promoting family food environments like shared family meals. Our findings contribute to previous research by reducing this gap in literature and may inform the design of future public health interventions aiming to promote family meals and reduce disparities in health from an early age.

The use of a validated and well‐known instrument to measure maternal mental health symptoms of depression and anxiety is a strength of the study. It is further a strength to include both breakfast and dinner and a research‐based frequency of regularity to encompass a health‐promoting practice of family meals. Also, the inclusion of relevant child and maternal correlates, as well as reporting results from two different time‐points with similar results, can be considered as strengths of the study.

However, there are also limitations that warrant discussion. The associations presented are correlational in nature and causality cannot be inferred. Longitudinal research is needed to provide additional evidence to support this relationship. The data are self‐reported, which may increase the risk of misreporting, however the number of participants made other approaches difficult. In addition, the low variability in the participants' socioeconomic status is a weakness of this study. The numbers of mothers living alone and mothers with low education were small, which may have resulted in an underestimation of socioeconomically patterned characteristics and could make it difficult to generalize from our findings. Further, despite including several child and maternal correlates, we cannot exclude residual or unmeasured confounding of the presented associations. Lastly, to increase the insight and understanding of how mothers with depression or anxiety experience having shared family meals and how to provide the best possible support, there is a need for qualitative studies.

## 5 CONCLUSION

Given the benefits and the importance of the family meal as a setting for child diet and dietary behaviors, it is important to identify potential modifiable barriers to having regular family meals. This is one of few studies to explore whether maternal mental health is associated with child participating in family meals during the first 2 years of life, a period where long‐lasting eating behaviors and the fundament for life‐long health are shaped. We found that maternal symptoms of anxiety and depression was associated with child having irregular family meals at both 12 and 24 months of age. Our findings support the importance of early identification of symptoms of anxiety and depression in mothers of infants and toddlers to provide the necessary support, including helping mothers to have positive interactions around food and eating with their children.

## AUTHOR CONTRIBUTIONS

All three authors contributed by the design and implementation of the original RCT. Christine Helle and Nina Cecilie Øverby contributed by the design and conception of the current study. Christine Helle collected the data, performed the analyzes, and drafted the paper. Elisabet Rudjord Hillesund and Nina Cecilie Øverby critically reviewed the manuscript and contributed to the interpretation of the findings. All the named authors have approved the submitted manuscript for publication.

## CONFLICT OF INTEREST STATEMENT

The authors declare no conflicts of interest.

## Supporting information

Supporting information.

## Data Availability

The data that support the findings of this study are available from the corresponding author upon reasonable request.
