# Supplementary material for: Maternal mental health is associated with children's frequency of family meals at 12 and 24 months of age
Source: Matern Child Nutr. 2023 Aug 18;20(1):e13552. doi: 10.1111/mcn.13552 (PMC10750025; doi:10.1111/mcn.13552)
Supplement: Supplementary file 1 — Supporting information. [file MCN-20-e13552-s001.pdf]

Table_4_Suppinfo.pdf:

Separate associations between, respectively, maternal symptoms of anxiety and maternal symptoms of depression and child having regular or irregular family meals at 12 and 24 months of age, OR (95% CI)

Table 4a Associations between maternal symptoms of anxiety and child having regular family meals at 12 and 24 months of age, OR (95% CI)

**Maternal and child characteristics**	**Regular family meals[1] 12 months (n = 270) n $_{tot}$ = 455**			**Regular family meals[1] 24 months (n = 171) n $_{tot}$ = 295**		
	**OR**	**95% CI**	**Sig.**	**OR**	**95% CI**	**Sig.**
**Maternal**						
Anxiety **(SCL-8 score)**	0.824	0.547 – 1.341	0.437	0.717	0.394 – 1.305	0.276
Age **(years)**	1.009	0.958 – 1.061	0.744	1.041	0.979 – 1.107	0.201
Education						
High *(college/university)*	1.000			1.000		
Low *(no college/university)*	0.968	0.552 – 1.700	0.911	0.996	0.487 – 2.035	0.991
Main activity						
Working	1.000			1.000		
At home	0.943	0.595 – 1.495	0.803	1.011	0.569 – 1.796	0.970
**0.970**						
Only child						
Yes	1.000			1.000		
No	0.581	0.380 – 0.890	**0.013**	0.916	0.557 – 1.505	0.728
Gender						
Girl	1.000			1.000		
Boy	1.291	0.874 – 1.906	0.199	1.540	0.955 – 2.482	0.076
Kindergarten						
Yes	1.000			1.000		
No	0.621	0.409 – 0.944	**0.026**	0.170	0.037 – 0.788	**0.024**

[1]Having breakfast and dinner with at least one adult eating the same meal five or more times per week.

Table 4b Associations between maternal depression and child having regular family meals at 12 and 24 months of age, OR (95% CI)

Maternal and child characteristics		Regular family meals[1] 12 months (n = 270) n $_{tot}$ = 455			Regular family meals[1] 24 months (n = 171) n $_{tot}$ = 295		
		**OR**	**95% CI**	**Sig.**	**OR**	**95% CI**	**Sig.**
**Maternal**							
**Depression (SCL-8 score)**		1.047	0.697 – 1.573	0.825	0.792	0.478 – 1.314	0.367
***Age* (years)**		1.010	0.960 – 1.063	0.693	1.042	0.979 – 1.108	0.194
Education							
	High *(college/university)*	1.000			1.000		
	Low *(no college/university)*	0.999	0.568 – 1.760	0.998	1.000	0.489 – 2.047	0.999
Main activity							
	Working	1.000			1.000		
	At home	0.944	0.595 – 1.496	0.805	0.998	0.561 – 1.775	0.995
**Child**							
	Only child						
	Yes	1.000			1.000		
	No	0.586	0.384 – 0.898	**0.014**	0.899	0.544 – 1.485	0.677
	Gender						
	Girl	1.000			1.000		
	Boy	1.292	0.875 – 1.908	0.198	1.529	0.949 – 2.461	0.081
	Kindergarten						
	Yes	1.000			1.000		
	No	0.609	0.400 – 0.927	**0.021**	0.170	0.037 – 0.786	**0.023**

Table 4c Associations between maternal anxiety and child having irregular family meals at 12 and 24 months of age, OR (95% CI)

**Maternal and child characteristics**	**Irregular family meals[1] 12 months (n = 270) n $_{tot}$ = 455**			**Irregular family meals[1] 24 months (n = 171) n $_{tot}$ = 295**		
	**OR**	**95% CI**	**Sig.**	**OR**	**95% CI**	**Sig.**
**Maternal**						
**Anxiety symptoms (SCL-8 score)**	2.137	1.186 – 3.850	**0.011**	2.619	1.221 – 5.620	**0.013**
***Age* (years)**	1.023	0.956 – 1.096	0.510	0.974	0.880 – 1.079	0.618
Education						
High *(college/university)*	1.000			1.000		
Low *(no college/university)*	1.179	0.557 – 2.498	0.666	0.680	0.241 – 1.917	0.466
Main activity						
Working	1.000			1.000		
At home	0.829	0.460 – 1.495	0.534	0.703	0.287 – 1.720	0.440
**Child**						
Only child						
Yes	1.000			1.000		
No	3.532	1.889 – 6.603	**≤0.001**	0.875	0.380 – 2.011	0.753
Gender						
Girl	1.000			1.000		
Boy	0.481	0.286 – 0.808	**0.006**	0.439	0.191 – 1.006	0.052
Kindergarten						
Yes	1.000			1.000		
No	1.073	0.621 – 1.854	0.800	2.010	0.242 – 16.716	0.518

Table 4d Associations between maternal depression and child having regular family meals at 12 and 24 months of age, OR (95% CI)

Maternal and child characteristics	Irregular family meals[1] 12 months (n = 270) n_{tot} = 455			Irregular family meals[1] 24 months (n = 171) n_{tot} = 295		
	**OR**	**95% CI**	**Sig.**	**OR**	**95% CI**	**Sig.**
**Maternal**						
**Depression symptoms (SCL-8 score)**	1.632	0.994 – 2.680	0.053	1.871	0.952 – 3.679	0.069
Age **(years)**	1.022	0.954 – 1.094	0.817	0.972	0.879 – 1.076	0.586
Education						
High *(college/university)*	1.000			1.000		
Low *(no college/university)*	1.203	0.568 – 2.547	0.630	0.678	0.241 – 1.910	0.462
Main activity						
Working	1.000			1.000		
At home	0.823	0.458 – 1.478	0.514	0.727	0.300 – 1.766	0.482
**Child**						
Only child						
Yes	1.000			1.000		
No	3.447	1.858	**≤0.001**	0.927	0.879 – 1.076	0.586
Gender						
Girl	1.000			1.000		
Boy	0.493	0.294 – 0.826	**0.007**	0.450	0.198 – 1.025	0.057
Kindergarten						
Yes	1.000			1.000		
No	1.067	0.617 – 1.845	0.817	2.006	0.244 – 16.495	0.517
